# Supplementary material for: The endosymbiont Spiroplasma poulsonii increases Drosophila melanogaster resistance to pathogens by enhancing iron sequestration and melanization
Source: mBio. 2024 Jun 28;15(8):e00936-24. doi: 10.1128/mbio.00936-24 (PMC11323552; doi:10.1128/mbio.00936-24)
Supplement: Supplemental material — Fig. S1-S6. [file mbio.00936-24-s0001.pdf]

Supplemental material for

The endosymbiont *Spiroplasma poulsonii* increases *Drosophila melanogaster* resistance to pathogens by enhancing iron-sequestration and melanization

Alexandra Hrdina[a, b, §], Marina Serra Canales[a, *, §], Aranzazu Arias-Rojas[a, c], Dagmar Frahm[a], Igor Iatsenko[a,#]

[a] Research group Genetics of host-microbe interactions, Max Planck Institute for Infection Biology, Charitéplatz 1, 10117 Berlin, Germany

[b] Humboldt-Universität zu Berlin, Faculty of Life Sciences, 10099 Berlin, Germany

[c] Department of Biology, Chemistry, and Pharmacy, Freie Universität Berlin, Berlin 14195, Germany

[*] Present address: College of Medical, Veterinary & Life Sciences, School of Molecular Biosciences, University of Glasgow, G12 8QQ, Glasgow, United Kingdom

[§] Alexandra Hrdina and Marina Serra Canales contributed equally to this work. Author order was determined on the basis of seniority.

# Corresponding author: Igor Iatsenko

Email: iatsenko@mpiib-berlin.mpg.de

Running title: Mechanisms of *Spiroplasma*-mediated host protection

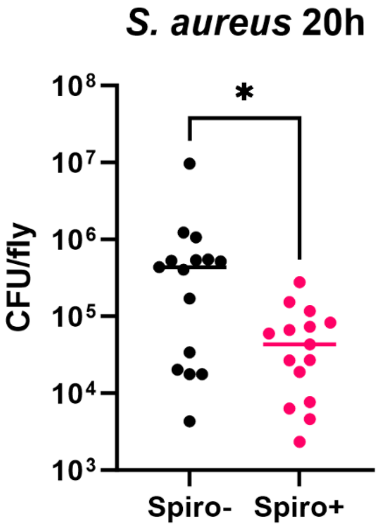

Figure S1. Effect of *Spiroplasma* on *S. aureus* proliferation in the host. Measurement of *S. aureus* burden at 20 hours post infection in *Spiroplasma*-free (Spiro-) and *Spiroplasma*-harbouring (Spiro+) flies. For cfu counts, each dot represents cfus from a pool of five animals, calculated per fly. The mean and SD are shown. Asterisks indicate statistical significance. $*P \leq 0.05$; $**P \leq 0.01$; $***P \leq 0.001$; $****P \leq 0.0001$; ns, nonsignificant, $P > 0.05$.

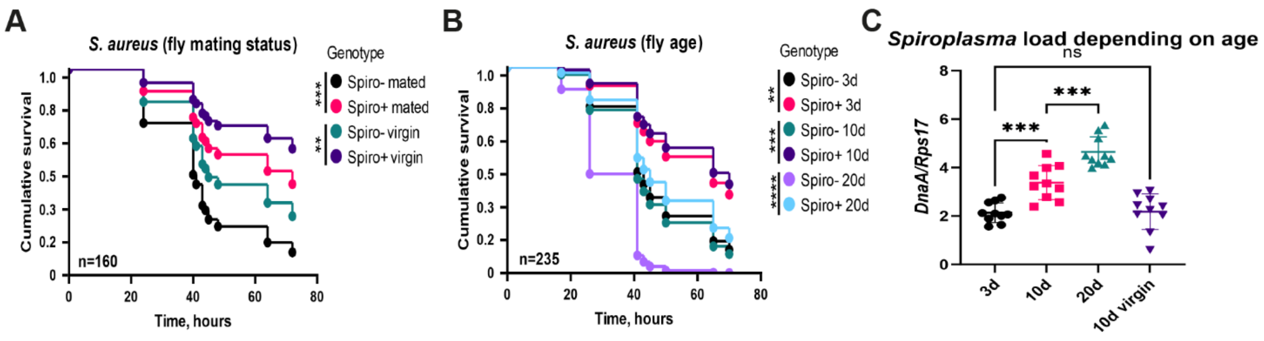

Figure S2. Effect of the *Drosophila* mating status and age on *Spiroplasma*-mediated defense. (A) Survival rates of *Spiroplasma*-free (Spiro-) and *Spiroplasma*-harbouring (Spiro+) *Oregon R* flies either mated or virgin after infection with *S. aureus.* (B) Survival rates of *Spiroplasma*-free (Spiro-) and *Spiroplasma*-harbouring (Spiro+) *Oregon R* flies of different age after infection with *S. aureus.* n = total number of flies in experiments. (C) Quantification of *Spiroplasma* titer by RT-qPCR in flies of various age. The mean and SD are shown. One-way Anova and Tukey's multiple comparisons test were applied. Asterisks indicate statistical significance. *P ≤ 0.05; **P ≤ 0.01; ***P ≤ 0.001; ****P ≤ 0.0001; ns, nonsignificant, P > 0.05.

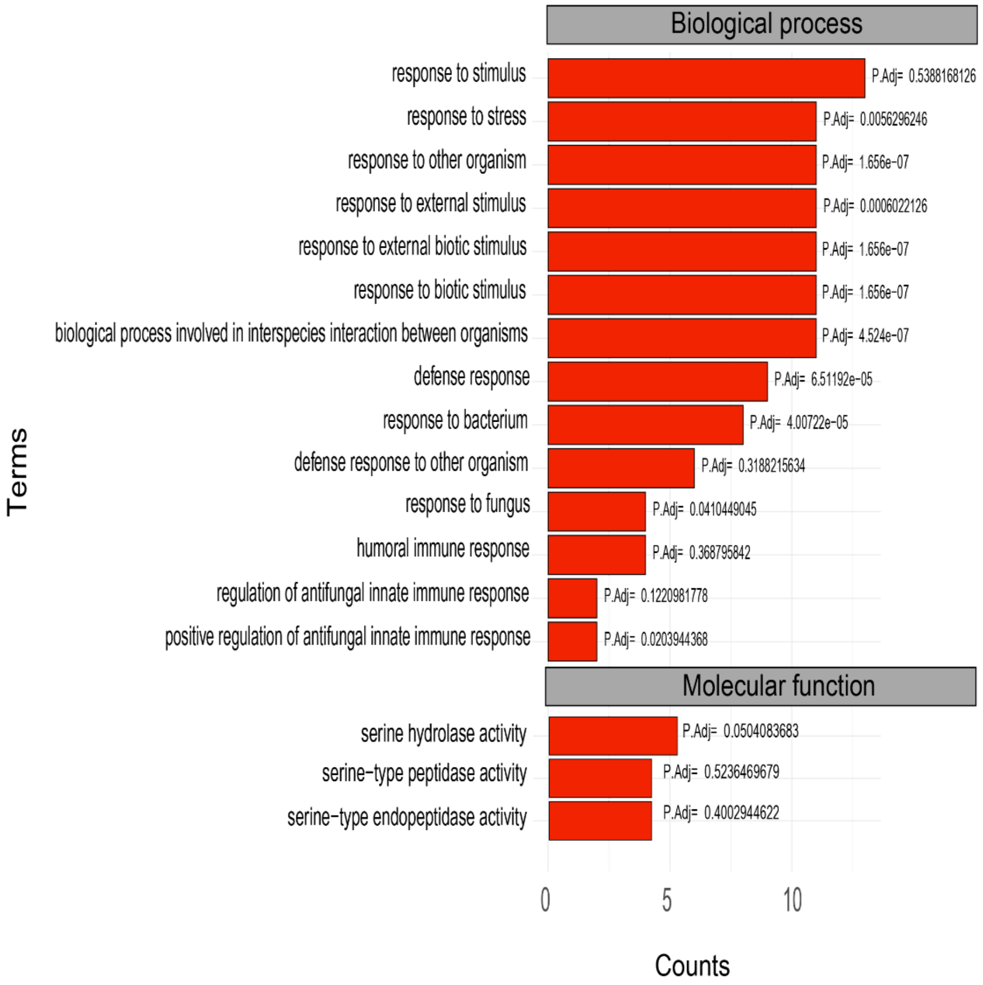

Figure S3. Functional GO annotation of genes upregulated in Spiro+ flies.

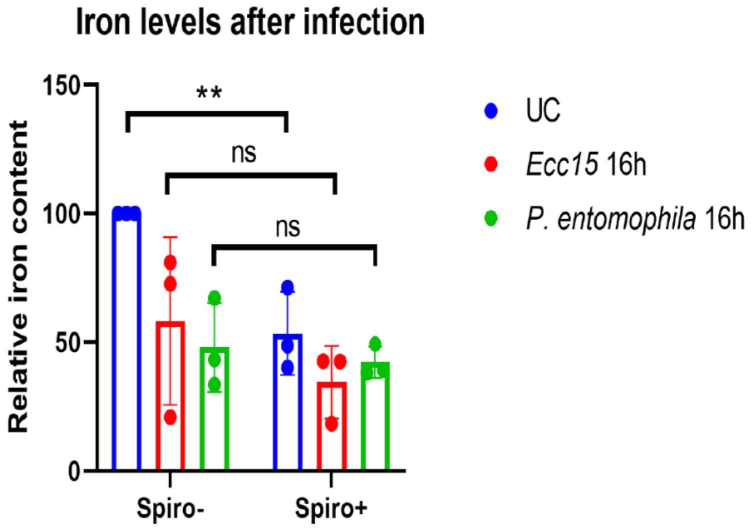

Figure S4. Effect of various pathogens on hemolymph iron level. Haemolymph iron content in Spiro- and Spiro+ uninfected flies and 16h after *Ecc15* or *P. entomophila* infection measured by the ferrozine assay. Iron content in uninfected Spiro- flies was set to 100 and all other values were expressed as a percentage of this value. The mean and SD of three independent experiments are shown. Asterisks indicate statistical significance. *P ≤ 0.05; **P ≤ 0.01; ***P ≤ 0.001; ****P ≤ 0.0001; ns, nonsignificant, P > 0.05.

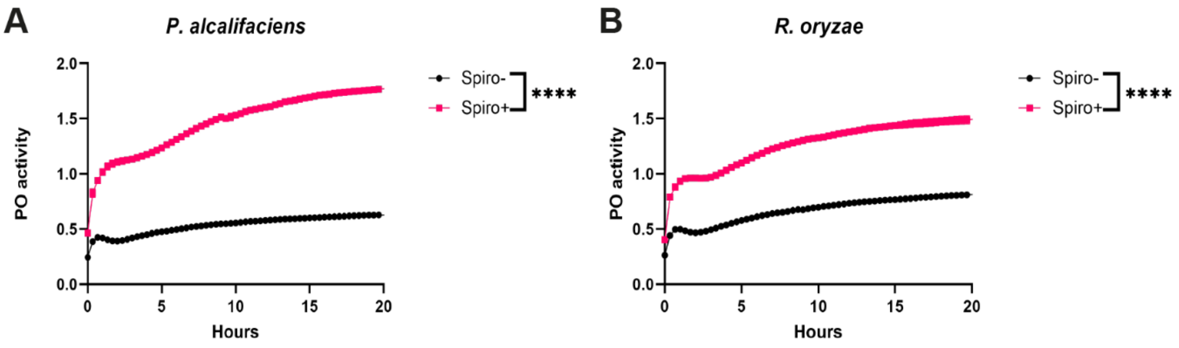

Figure S5. *Spiroplasma*-harbouring flies exhibit enhanced melanization independently of the infecting pathogen. (A, B) Hemolymph phenoloxidase (PO) activity 3 h after *P. alcalifaciens* (A) or *R. oryzae* (B) infection in *Spiroplasma*-free (Spiro-) and *Spiroplasma*-harbouring (Spiro+) *Oregon R* flies measured by the L-DOPA assay over 20 h period. One representative experiment is shown.

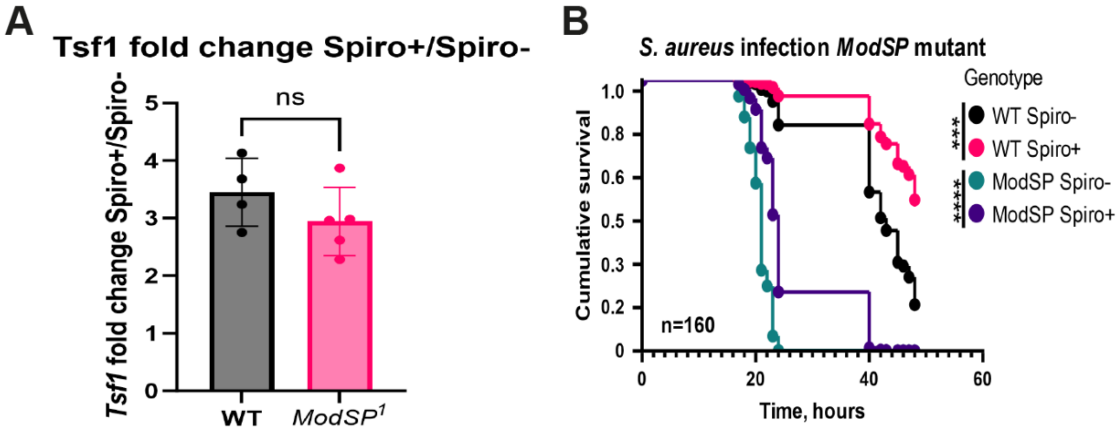

Figure S6. *ModSP* is not required for *Spiroplasma*-mediated protection. (A) RT-qPCR showing differences in the fold change of *tsf1* gene expression induced by *Spiroplasma* in wild-type and $ModSP^1$ mutant flies. The mean and SD of 5 independent experiments are shown. (B) Survival rates of *Spiroplasma*-free (Spiro-) and *Spiroplasma*-harbouring (Spiro+) wild-type and $ModSP^1$ mutant flies after infection with *S. aureus*. n = total number of flies in experiments. Asterisks indicate statistical significance. *P ≤ 0.05; **P ≤ 0.01; ***P ≤ 0.001; ****P ≤ 0.0001; ns, nonsignificant, P > 0.05.

Table S1. List of genes differentially-expressed between *Spiroplasma*-harboring and *Spiroplasma*-free 10d-old *Oregon R* female flies.
